# A case report of multiple histological subtypes ameloblastoma with millet-like calcification

**DOI:** 10.3389/fonc.2026.1801359

**Published:** 2026-04-23

**Authors:** Xihai Gao, Jinkui Zhao, Xiangyu Ma, Bao Zhong, Mingru Fan, Jingchao Han

**Affiliations:** 1 College of Medical Technology, Binzhou Polytechnic, Binzhou, Shandong, China; 2 Department of Medical Imaging, Central Laboratory of Jinan Stomatological Hospital, Jinan, Shandong, China; 3 Department of Pathology, Experimental Diagnosis Department, Jinan Jinyu Medical Laboratory Center Co., Ltd., Jinan, Shandong, China

**Keywords:** ameloblastoma, calcification, CBCT, multiple histological subtypes, pathology

## Abstract

Interstitial calcification in Ameloblastoma (AM) with multiple histological subtypes is extremely rare. Notably, no reports exist regarding this feature in unicystic AM. Here, we report a case of a 20-year-old female who presented with a one-month history of chewing pain in the left mandibular posterior region. Cone-beam computed tomography (CBCT) revealed impacted second and third molars in the left mandible. A well-defined cystic lesion surrounded the crowns of these molars, accompanied by partial resorption of the bone plate and areas of bone destruction on the lingual aspect. Multiple millet-like calcifications were observed within the cyst. The lesion involved the root trunk of the first molar but spared the apex. Treatment consisted of curettage and extraction of the impacted teeth. Pathological examination confirmed AM with multiple histological subtypes and interstitial calcification. The Ki-67 proliferation index was approximately 5%. One year post-surgery, the bone structure in the operative area had regenerated well, with no signs of recurrence. This case suggests that AM can present as a mixed form of multiple histological subtypes with millet-like calcifications in the tumor stroma. Routine tumor curettage can yield satisfactory results. This report provides a valuable reference for the diagnosis and treatment of unicystic AM characterized by multiple histological subtypes and millet-like calcification.

## Introduction

Ameloblastoma (AM) is a relatively common odontogenic epithelial tumor, with an incidence rate of approximately 0.92 cases per million people annually (1, 2). In the updated 2022 World Health Organization (WHO) classification of head and neck tumors, AM is categorized into several main types: conventional (formerly known as multicystic or solid), unicystic (6%), extraosseous/peripheral (2%), and metastasizing (1%) (3).

Histologically, AM displays considerable heterogeneity in both tissue architecture and cellular morphology. It comprises six main histological subtypes: follicular, plexiform, acanthomatous, granular cell, basal cell, and desmoplastic. Among these, the follicular and plexiform variants are the most prevalent (3, 4).

Reports of AM exhibiting multiple coexisting histological subtypes are scarce, and cases featuring millet-like calcification are exceptionally rare. Herein, we report a case of unicystic AM in a 20-year-old female, characterized by multiple coexisting histological subtypes and stromal millet-like calcification. Notably, the tumor encompassed two impacted teeth, a presentation that offers valuable insights for improving clinical diagnosis and therapeutic strategies.

## Case description

This case report was approved by the Ethics Committee of Jinan Stomatological Hospital (No. JNSKQYY-2025-003).

**Figure 1:**
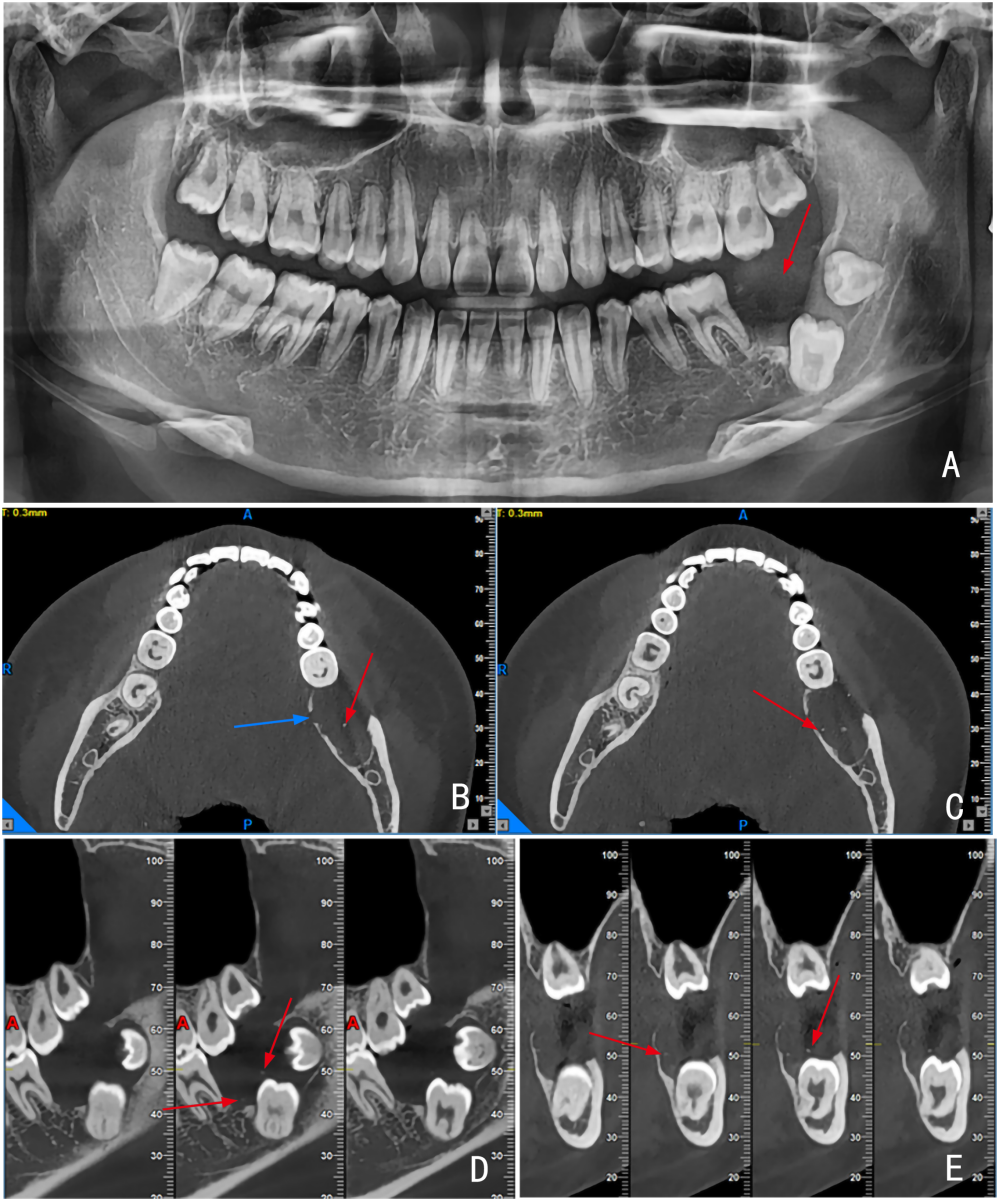
Preoperative orthopantomogram showing a well-defined cystic lesion surrounding the crowns of the second and third molars [image **(A)**, red arrow]. CBCT images in axial [images **(B)** and **(C)**], sagittal [image **(D)**], and coronal [image **(E)**] views. The lesion caused partial resorption of the buccal and lingual cortical plates, with uneven thickness observed in the lingual plate [image **(B)**, blue arrow]. Multiple millet-like high-density calcifications were scattered within the lesion [images **(B–E)**, red arrows].

A 20-year-old female presented with a one-month history of chewing pain in the left mandibular posterior region. An orthopantomogram obtained at a local hospital revealed two impacted teeth in the left mandible surrounded by a cystic lesion [**Figure 1** (image A)]. The preliminary diagnosis was a dentigerous cyst. The patient was subsequently referred to our institution, where cone-beam computed tomography (CBCT) was performed to refine the diagnosis, and surgical intervention was recommended.

Clinical examination revealed that the left mandibular second and third molars were not visible. The overlying gingiva appeared dark brown. The lesion felt soft and fluctuant on palpation.

CBCT imaging [**Figure 1** (images B–E)] revealed the left mandibular second molar was vertically impacted at a low position, and the left mandibular third molar was horizontally impacted with a forward inclination. A well-defined cystic lesion was located around the crowns of the second and third molars, with partial resorption of the buccal and lingual bone plates (images B and C). A zone of dense bone destruction was visible on the lingual plate, with uneven thickness of the bone walls. Multiple millet-like calcifications were seen in the cyst (images B, C, D and E), and the distribution was uneven. The lesion involved the distal root body of the first molar, but not the root tip. There was no significant widening of the periapical membrane (image D). A preliminary clinical diagnosis was made of cystic tumor-like lesions.

**Figure 2:**
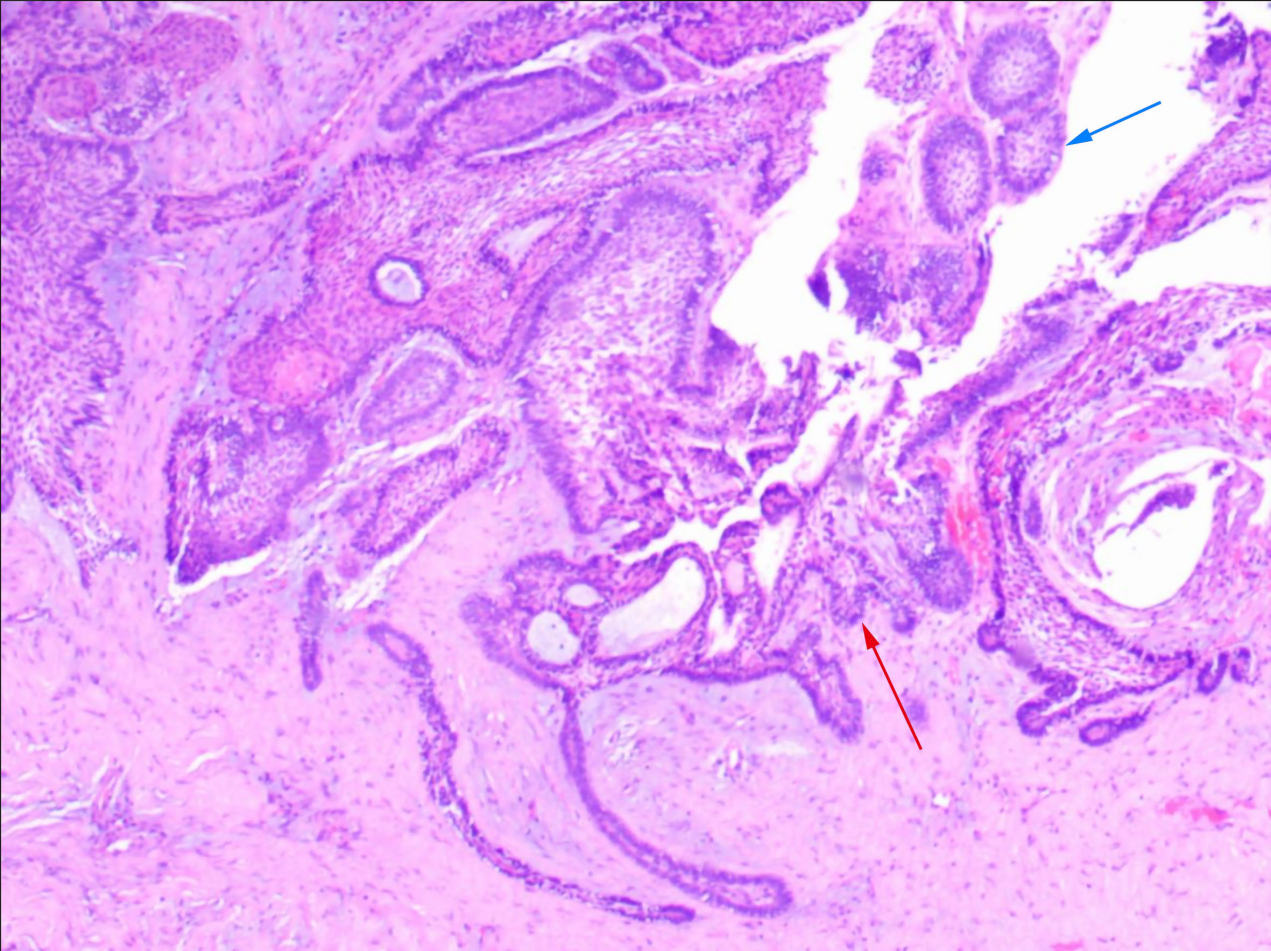
Intraoperative frozen section analysis. The image reveals the morphological features of follicular (blue arrow) and plexiform (red arrow) AM (HE × 40).

Intraoperative frozen section analysis suggested AM (**Figure 2**). Guided by the CBCT findings, enucleation and curettage of the cystic lesion in the left mandible, along with extraction of the impacted teeth, were performed. The surgical specimen was subsequently submitted for final histopathological analysis.

**Figure 3:**
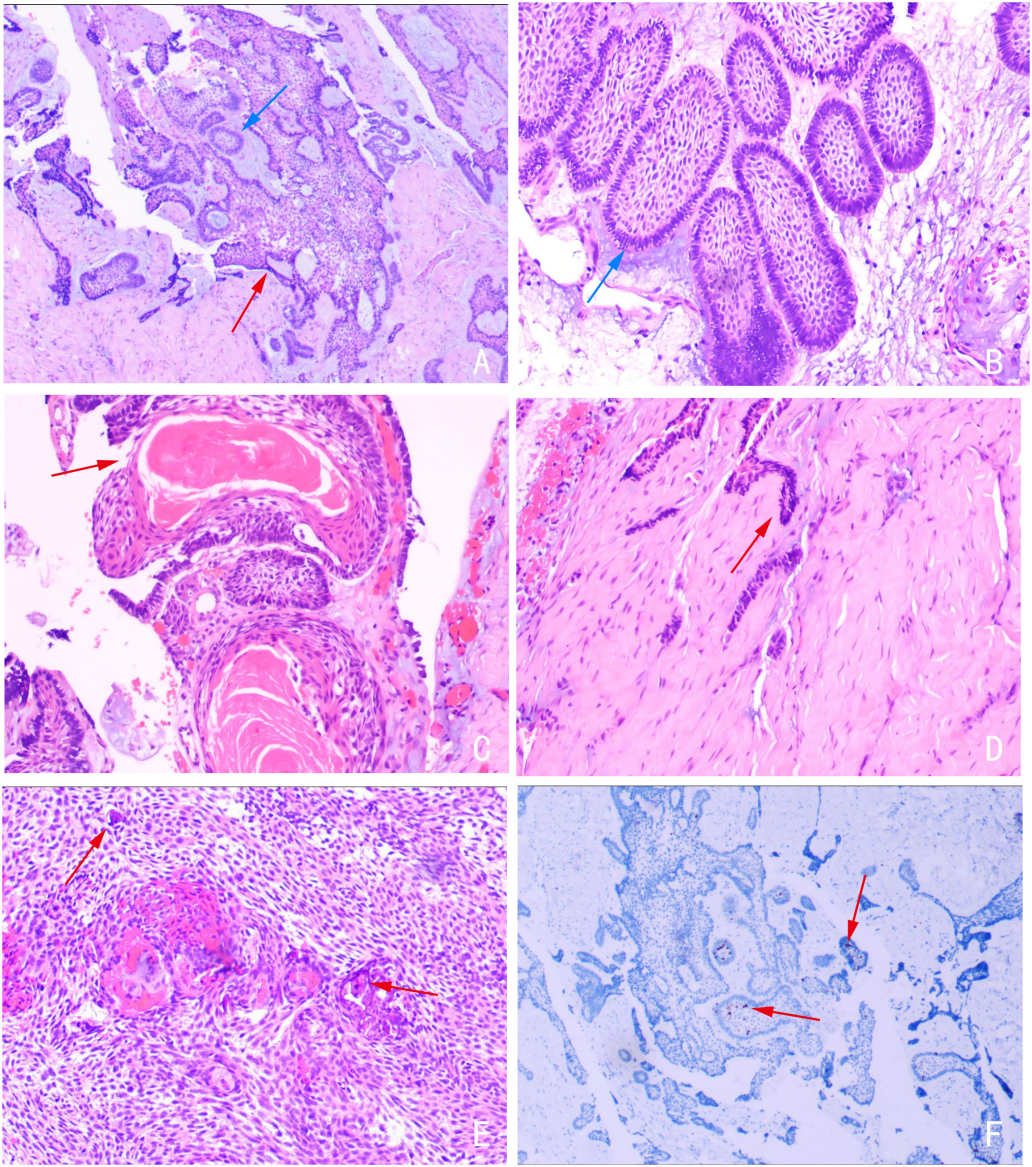
Postoperative histopathology of the resected tumor revealing multiple subtypes of AM. Image **(A)** Plexiform type (red arrow) and Follicular type (blue arrow); **(B)** Follicular type (blue arrow); **(C)** Acanthomatous type showing keratin pearls (red arrow). **(D)** Basal cell type (red arrow). **(E)** Calcification foci within the tumor stroma (red arrow). **(F)** Ki-67 immunohistochemical staining demonstrating a labeling index of approximately 5% (red arrow). [HE stain for images **(A–E)**; Immunohistochemistry for image **(F)**].

The entire surgical specimen was examined using serial sections. Histopathological examination (**Figure 3**) revealed the following features: (1) Plexiform type: tumor epithelium proliferating in a net-like pattern of epithelial cords, surrounded by a layer of cuboidal or columnar cells (image A); (2) Follicular type: solitary epithelial islands composed of centrally located polygonal, stellate-reticulum-like cells, surrounded by a peripheral layer of columnar epithelial cells (image B); (3) Acanthomatous type: squamous metaplasia within epithelial islands with the formation of keratin pearls (image C); (4) Basal cell type: densely packed clusters of small, uniform tumor cells exhibiting a palisading appearance (image D); (5) Calcification foci were observed within the tumor stroma (image E). The Ki-67 labeling index was approximately 5% (image F). In summary, this case of AM exhibited multiple histological subtypes accompanied by stromal calcification.

## Follow-up and outcomes

**Figure 4:**
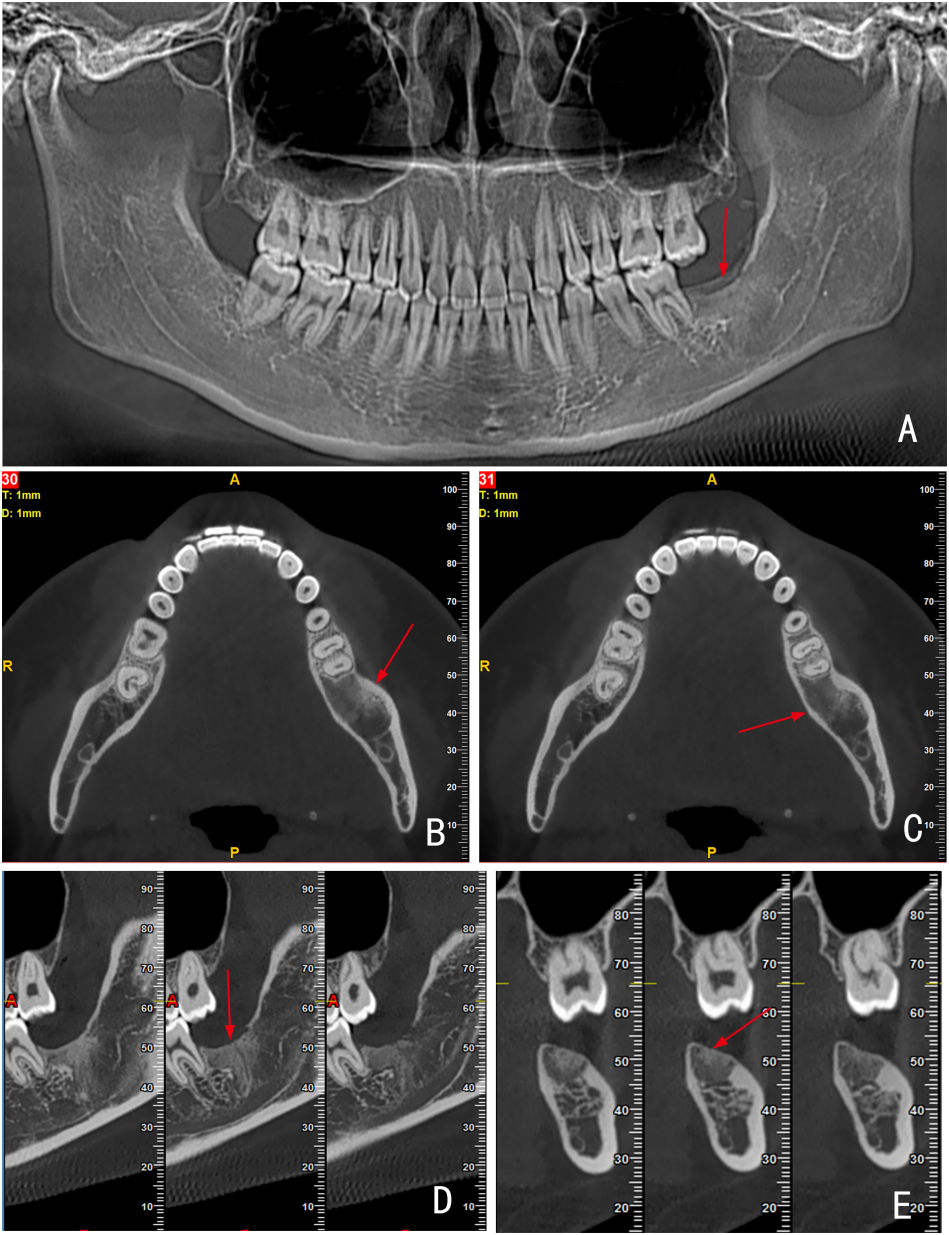
CBCT images obtained at the one-year follow-up after tumor resection. Image **(A)** Panoramic reconstruction; **(B, C)** Axial views; **(D)** Sagittal view; **(E)** Coronal view. All images demonstrate complete bone remodeling at the surgical site (red arrows).

At the one-year postoperative follow-up, the surgical site demonstrated favorable bone regeneration with no radiographic signs of recurrence. Specifically, the alveolar bone surrounding the distal root of the adjacent mandibular first molar exhibited complete remodeling (**Figure 4**). The patient reported no discomfort at the surgical site and expressed satisfaction with the treatment outcome.

## Discussion

### AM imaging manifestations and calcification analysis

The imaging diagnosis of AM is well-established, particularly for the conventional solid/multicystic type, which typically presents as cystic bone destruction of varying sizes. While AM associated with impacted teeth is typically of the unicystic subtype (5, 6), the present case involved two impacted teeth embedded within the tumor. This is an extremely rare presentation that poses a significant risk of misdiagnosis as a dentigerous cyst. However, the presence of small cystic destructive areas along the superior aspect of the lingual bone plate, combined with intra-tumoral calcification foci, distinguishes this lesion from a dentigerous cyst, as these features are characteristically absent in the latter.

A PubMed search identified five relevant reports comprising six cases of calcification, all confirmed as dystrophic calcification. This type of calcification results from irreversible degeneration and local necrosis of AM cells, leading to disrupted calcium homeostasis and subsequent deposition of calcium salts in the tissue (7–11). In five of these reported cases, calcification was identified solely via histopathological examination. Although calcification was detected radiographically in the cases reported by Kang et al. (11) and in the present case, only the present case involved concomitant impacted teeth (**Table 1**).

**Table 1:** AM with calcification: a chronological review of case reports (1958–2025).

Author (year)	Age	Gender	Clinical features	Imaging findings (X-ray/CBCT)	Histopathological finding	Treatment and recurrence
Pindborg JJ et al. (1958)	75	Female	Painless fluctuant swelling in the right mandibular third molar region and ascending ramus, with a draining sinus tract.	X-ray shows an oval radiolucent area.	Acanthomatous AM with squamous cell metaplasia and calcification.	Underwent a second marsupialization.
	24	Male	Small swelling in the left mandibular second premolar and first molar region.	X-ray shows a large, well-demarcated radiolucent area in the left mandible.	Follicular AM, showing calcification at different stages of keratin pearls formation.	Resection of the mandible from canine to second molar with rib graft implantation. No recurrence after 4 years of follow-up.
Siar CH et al. (1991)	35	Male	Right maxillary swelling persisted for 5 years, slow-growing, firm and painless.	X-ray shows a radio-opaque mass extending into the right maxillary sinus.	Acanthomatous AM, showing keratin pearls formation and dystrophic calcification.	Right hemimaxillectomy and obturator placement. No recurrence after 3 years of follow-up.
Kim JD et al. (1995)	63	Male	Not reported.	CBCT shows a unilocular calcified lesion (right maxillary antrum) with enhancing alveolar mass.	Acanthomatous AM, showing keratin pearls formation and dystrophic calcification within the epithelial islands.	Not reported.
Lascane NA et al. (2014)	56	Male	Painless lesion in the vestibular gingival region between the mandibular left incisors, persisted for 10 months.	X-ray and CBCT show only superficial erosion of the underlying bone crest.	Acanthomatous AM, several basophilic areas compatible with dystrophic calcification.	Extended excision on the border of the former lesion. No signs of local recurrence 1 year after treatment.
Kang BC et al. (2020)	22	Female	Hard swelling on the right mandible.	X-ray and CBCT show multilocular radiolucencies with internal calcification foci in the right mandible.	Acanthomatous AM with dystrophic calcification.	Segmental mandibulectomy with free fibula flap reconstruction. No signs of local recurrence.
Present Case	20	Female	Presented with chewing pain in the left mandibular posterior tooth region, persisted for 1 month.	CBCT shows a well-defined cystic lesion around second and third molar crowns with multiple millet-like calcifications.	Multiple histological subtypes AM with interstitial calcification.	Treated with curettage and extraction of the impacted teeth. No recurrence after 1 year.

AM, Ameloblastoma; CBCT, Cone Beam Computed Tomography.

The calcification patterns observed on CBCT in this case correlated well with histopathological findings, suggesting that millet-like calcification can occur in AM. In contrast, calcification in calcifying odontogenic cysts (COC) typically occurs within the cyst wall rather than the lumen and often appears patchy, a feature useful for differentiation. Similarly, calcifying epithelial odontogenic tumors (CEOT) may exhibit calcification and are frequently associated with unerupted teeth, with calcifications typically located adjacent to the crowns (12). Since the present case involved unerupted teeth with calcifications located in the pericoronal region, there was a risk of misdiagnosis as CEOT. However, multifocal small cystic bone destruction is rarely observed in COC or CEOT. This radiographic sign not only aids in differential diagnosis but also indicates the invasive nature of the tumor, suggesting that a more aggressive surgical approach is required compared to that for ordinary cystic lesions.

### Pathological analysis of multiple histological subtypes AM

Currently, there are few reports in the literature of multiple subtypes coexisting in the primary diagnosis of the same AM. Specifically, acanthomatous type usually occurs in follicular AM, characterized by squamous metaplasia within the tumor’s epithelial islands, occasionally accompanied by keratin pearl formation (13). Arushi et al. (13) reported a rare case of acanthomatous transformation occurring in a plexiform AM. This case highlights the potential for squamous metaplasia in plexiform AM, manifesting with acanthomatous features. Histologically, AM exhibiting acanthomatous features must be differentiated from squamous cell carcinoma and other acanthomatous odontogenic lesions. The presence of typical histological components of follicular or plexiform AM is crucial for a definitive diagnosis. The basal cell variant is an exceptionally rare subtype of AM. Histologically, the tumor epithelium forms dense clusters or cords; the cells are small and uniform, notably lacking differentiation into stellate reticulum-like cells. Consequently, differential diagnosis should include basal cell carcinoma and adenoid cystic carcinoma (4). Of the seven cases documented in previous literature (7–11) and the current study, six were of the acanthomatous subtype and one was follicular. This distribution suggests that the acanthomatous subtype may be more frequently associated with calcification, implying that interstitial calcification could be a characteristic feature of this variant.

Regarding biological behavior, Schoinohoriti et al. (14) reported that the coexistence of multiple histological subtypes in AM does not necessarily increase tumor invasiveness. However, Purwoyudho et al. (15) compared the expression of Ki-67 in 24 cases of AM, finding that the average Ki-67 expression value for 8 cases of plexiform and follicular mixed subtypes was 45.22, for 4 cases of follicular subtype was 40, and for 12 cases of plexiform subtype was 31.08. The Ki-67 proliferation index serves as a marker of tumor aggressiveness; elevated indices correlate with poorer prognosis and higher recurrence rates (16). In the present study, three representative high-power fields (400×) were selected to calculate the average percentage of Ki-67-positive cells. The results showed an overall Ki-67 positivity rate of approximately 5%, with the plexiform subtype exhibiting a slightly higher index than other subtypes. This value is significantly lower than that reported by Purwoyudho et al. (15). The relatively low proliferation level may be attributed to the nature of the cases in this study, which involved unicystic AM in younger patients with minimal cellular atypia. In contrast, the cohort studied by Purwoyudho et al. (15) primarily consisted of solid/multicystic AM.

### Treatment of AM

Literature reports indicate that the recurrence rate of AM following radical resection ranges from 15% to 25%, whereas conservative management is associated with significantly higher recurrence rates of 75% to 90% (4, 14). In contrast, unicystic AM typically affects adolescents and resembles odontogenic cysts in its well-circumscribed nature and less aggressive clinical behavior. Consequently, it is typically managed with conservative surgical approaches, such as curettage or decompression, yielding favorable prognoses and low recurrence rates (approximately 10%) (4). Radical resection is primarily reserved for cases where complete tumor removal cannot be achieved via conservative methods, or for recurrent and aggressive variants. Such procedures are associated with significant morbidity, a higher risk of complications, and often necessitate complex postoperative reconstruction. Furthermore, for cases where surgery is contraindicated or the tumor is deemed inoperable, adjuvant therapies including radiotherapy and targeted molecular agents have been investigated (17).

Among the six cases of calcified AM reported between 1958 and 2025 (16), five underwent radical surgery, with no postoperative recurrence observed (**Table 1**). In the present case, the tumor was relatively large and encompassed two impacted molars. Radiographic examination revealed no significant multilocular changes; the lesion exhibited well-defined boundaries and showed no infiltration into the surrounding soft tissues. In accordance with the patient’s preference, conservative surgery was performed, consisting of thorough tumor curettage and extraction of the impacted teeth. Healthy mandibular tissue and the adjacent first molar were preserved to the greatest extent possible to maintain mandibular continuity, masticatory function, and facial aesthetics. Postoperative follow-up demonstrated favorable bone regeneration at the surgical site, meeting the criteria for dental implant restoration, while the adjacent first molar remained asymptomatic. Late recurrence of AM remains a well-documented clinical concern. As the current follow-up duration for this case is limited to one year, extended surveillance is warranted.

Zhu et al. (18) reported a significantly higher prevalence of BRAF V600E mutations in mandibular AMs [92% (59/64)] compared to maxillary AMs [40% (10/25)]. Conversely, SMO mutations were identified in 20% (5/25) of maxillary AMs but only 3.1% (2/64) of mandibular cases. Notably, BRAF and SMO mutations exhibited mutual exclusivity. For patients with BRAF V600E (+) or SMO (+), conservative surgical treatment has a higher recurrence rate than patients with BRAF V600E (-) or SMO (-); while when using radical surgical treatment, there is no significant difference between the two. Clinically, these findings suggest that the optimal surgical approach should be determined based on BRAF V600E and SMO mutation status (19). Furthermore, Silveira et al. (20) reported the prevalence of the BRAF V600E immunoexpression may suggest the feasibility of utilizing BRAF-targeted therapy for AM with this mutation.

## Conclusion

AM can be mixed with multiple histological subtypes, and millet-like calcification can also occur in tumor stroma. With the widespread adoption of CBCT in dental and maxillofacial imaging, the detection rate of calcification in AM is likely to rise, warranting heightened clinical awareness. Notably, despite the presence of mixed histological subtypes and interstitial calcification, this unicystic AM did not exhibit increased invasiveness. Adjacent teeth with uninvolved root apices and preserved pulp vitality can be successfully retained. Conventional tumor curettage can achieve satisfactory treatment results. This case serves as a valuable reference for the diagnosis and management of unicystic AM featuring mixed histological subtypes and millet-like calcification. Given that the current cases of AM with interstitial calcification and mixed multiple pathological subtypes are still very limited, it is recommended that large-sample, multi-center, prospective cohort studies be conducted to clarify the biological mechanism underlying low proliferative activity and its clinical prognostic value.

## Data Availability

The original contributions presented in the study are included in the article/supplementary material. Further inquiries can be directed to the corresponding authors.
